# TopicalPdb: A database of topically delivered peptides

**DOI:** 10.1371/journal.pone.0190134

**Published:** 2018-02-12

**Authors:** Deepika Mathur, Ayesha Mehta, Priyanka Firmal, Gursimran Bedi, Charu Sood, Ankur Gautam, Gajendra P. S. Raghava

**Affiliations:** 1 Bioinformatics Centre, CSIR-Institute of Microbial Technology, Chandigarh, India; 2 Computational Biology, Indraprastha Institute of Information Technology, New Delhi, India; International Centre for Genetic Engineering and Biotechnology, INDIA

## Abstract

TopicalPdb (http://crdd.osdd.net/raghava/topicalpdb/) is a repository of experimentally verified topically delivered peptides. Data was manually collected from research articles. The current release of TopicalPdb consists of 657 entries, which includes peptides delivered through the skin (462 entries), eye (173 entries), and nose (22 entries). Each entry provides comprehensive information related to these peptides like the source of origin, nature of peptide, length, N- and C-terminal modifications, mechanism of penetration, type of assays, cargo and biological properties of peptides, etc. In addition to natural peptides, TopicalPdb contains information of peptides having non-natural, chemically modified residues and D-amino acids. Besides this primary information, TopicalPdb stores predicted tertiary structures as well as peptide sequences in SMILE format. Tertiary structures of peptides were predicted using state-of-art method PEPstrMod. In order to assist users, a number of web-based tools have been integrated that includes keyword search, data browsing, similarity search and structural similarity. We believe that TopicalPdb is a unique database of its kind and it will be very useful in designing peptides for non-invasive topical delivery.

## Introduction

Over the last decade, peptides have emerged as an important class of therapeutics for the treatment of many diseases like cancer, skin infections or autoimmune diseases due to their high specificity and potency[1]. In the year 2012 alone, six peptide-based drugs have been approved by food and drug administration (FDA), which reflects the potential of the peptides as a therapeutic entity[2]. Despite the significant potential of peptide-based drugs, one of the major concerns with these drugs is the route of administration, which has a significant impact on the therapeutic outcome of a drug.

Peptide-based drugs are generally not administered orally because they are degraded extensively by proteases in the gastrointestinal (GI) tract before they reach circulation[3]. Though the parenteral route is the most common mode of administering these drugs and since it is a painful and invasive method, development of alternative non-invasive topical delivery formulations is the need of the hour. Therefore, considerable efforts have been made over the last few years to design and develop non-invasive and less painful routes for the administration of peptide-based drugs [3–5]. Routes on which significant attention has been paid is the topical delivery through skin[6, 7], eyes [8], and nose[9, 10], which present attractive and convenient alternative as they by-pass the first pass hepatic metabolism. Since they are easy to use, non-invasive and painless, they provide better patient compliance.

Topical delivery through skin looks very attractive, but at the same time, it is very challenging. The main obstacle in topical delivery through the skin is posed by the outermost dead layer of the epidermis *i*.*e*. stratum corneum (SC), which prevents molecules from diffusing readily[7]. Therefore, considerable efforts have been made to enhance the permeability of SC. Chemical modifications of peptides like the addition of lipophilic moieties produce analogs that show increased stability and skin permeability. Conjugation to CPPs and presence of enhancers in formulations has shown to enhance the penetration of peptides [11] through these topical routes.

Transmucosal nasal drug delivery has also emerged as an important field of drug delivery technology[12]. Avoidance of hepatic first-pass metabolism, as well as high vascularity, and large surface area makes the transmucosal nasal drug delivery more attractive and popular. Over the last decade, a wide variety of peptide therapeutics has been administered intranasally for topical, systemic and central nervous system action [13–15].

Similarly, ocular delivery is another route of drug administration, which is increasing gaining scientific attention, as it is safe and non-invasive and may maintain drug levels in tissues[16]. However, drug delivery through the eye is very challenging and restricted by various precorneal, dynamic and static ocular barriers. In order to overcome these limitations, in the past, many novel formulations with peptides have been developed and evaluated[17, 18].

Despite the significant importance of alternative non-invasive methods like topical delivery of peptides, to date, no heed has been paid to develop a dedicated repository of topically administered peptides. This information is very important but it is scattered in the literature and thus very difficult to access. In order to facilitate the scientific community engaged in developing peptide drug delivery, we have made an attempt to collect and compile all this scattered information related to topical delivery of peptides and developed a repository-TopicalPdb. TopicalPdb will be helpful for researchers working in the field of peptide therapeutics.

## Methods

### Data collection

In order to provide comprehensive information on topically delivered peptides, we have performed a systematic search in PubMed using a combination of keywords- "topical[Title/Abstract] AND peptide[Title/Abstract]" and "peptide[Title/Abstract] AND penetrating[Title/Abstract] AND (skin[Title/Abstract] OR dermis[Title/Abstract] OR dermal[Title/Abstract] OR eye[Title/Abstract] OR ocular[Title/Abstract] OR nasal[Title/Abstract] OR nose[Title/Abstract])" in the query. These searches ended in around 1000 research articles as on July 2016 giving information of topically administered peptides, which include skin, eye, and nose. After careful readings, the information of such peptides like length, sequence, origin of peptides and other relevant experimental information like N-terminal modifications, C-terminal modifications, mechanisms, assay, assay conditions and tissue sample was collected manually from 135 articles. TopicalPdb finally contains total 657 entries.

### Database architecture

**Fig 1:**
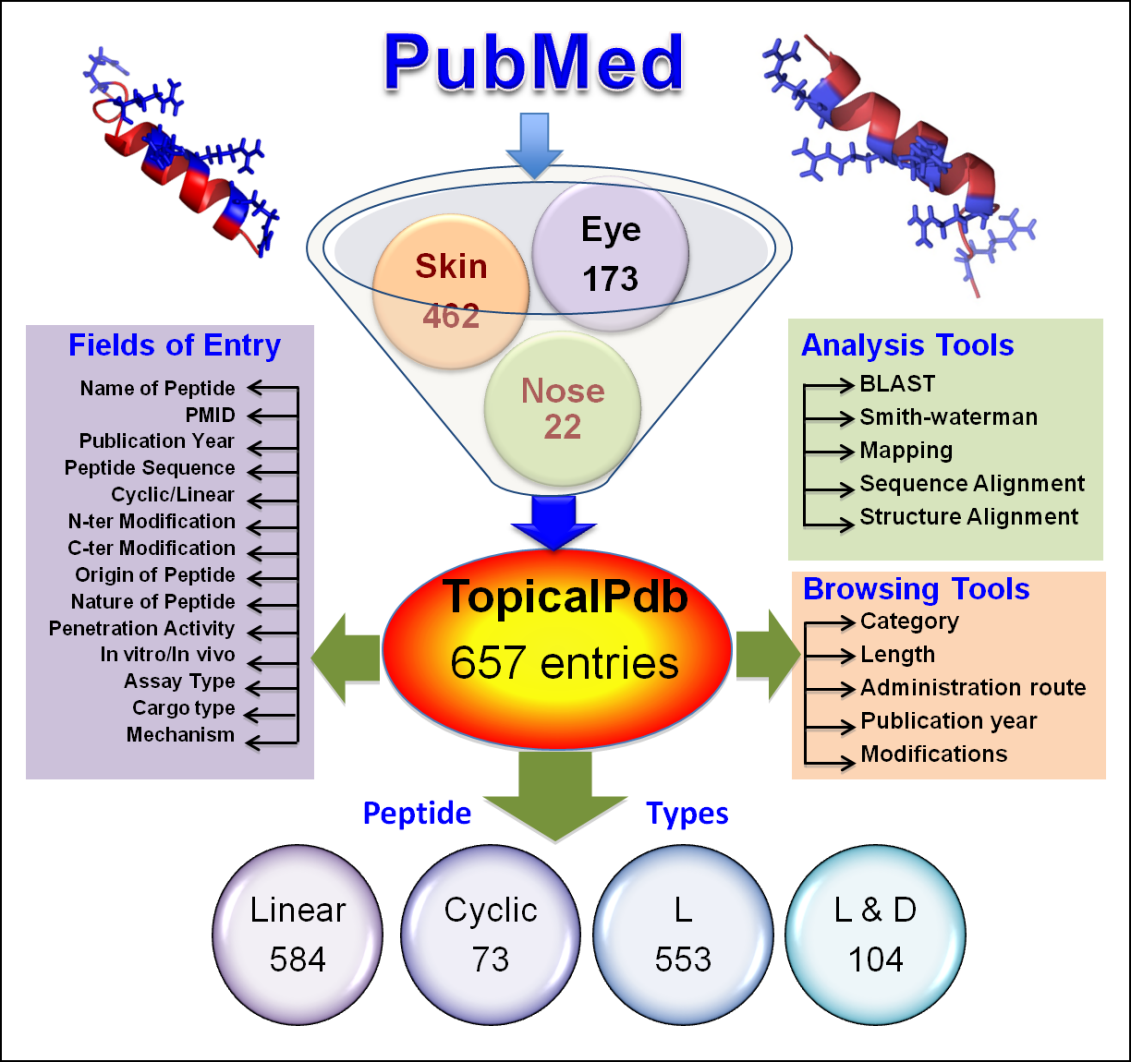
Architecture of TopicalPdb.

All the data was collected and compiled using MySQL. The database was launched using Apache HTTP server on Linux platform. We used HTML, PHP, JAVA to improve the web interface. Database architecture is shown Fig 1.

### Database content

The primary information on topically delivered peptides was manually curated from research articles and consists of various fields such as PubMed ID, year, sequence, name of peptide, length, N-terminal modification, C-terminal modification, chirality, chemical modification, origin of peptide, biological property of peptide, mechanism, cargo sequence or structure, name of cargo, assay types, tissue permeability, incubation, concentration, *in vivo*/ in vitro/ *ex vivo* systems and tissue sample. All this information was compiled systematically. For a better understanding of the effect of experimental conditions on the permeation ability of the peptides, we made multiple entries of the same peptide if the permeability was studied under different conditions like change in pH of formulation, concentration of the peptide, cargo attached to the peptide, tissue sample used during the assay, etc.

For the rational designing of topically administered peptides, it is imperative to understand their structure. In TopicalPdb, we have maintained the tertiary structure and SMILES of all peptide entries as secondary information. Firstly, all sequences were searched and mapped on PDB[19]. 58 exact sequence matches obtained were assigned structures as given in PDB. For peptides, whose identical sequences were not available in PDB, we used PEPstrMOD[20] to predict their structures. PEPstrMOD was used to predict the structure of 217 sequences of peptides with natural and modified residues with the length between 5 and 25. A total 69 peptide sequences had less than 5 residues and could not be predicted using PEPstrMOD. For such cases, dihedral angles were taken as 180. The structure obtained after energy minimization and molecular dynamic simulation was regarded as the final structure. The structures of natural peptides with length >40 were assigned using I-TASSER[21]. Open Babel[22] was deployed for the conversion of 3D PDB structures to 2D SMILES notation.

## Results and discussion

### Implementation

A user-friendly web-interface has been designed to query the database. For data searching and analysis, various tools have been integrated into the database. The brief description of these tools is as follows:

#### Searching tools

**Fig 2:**
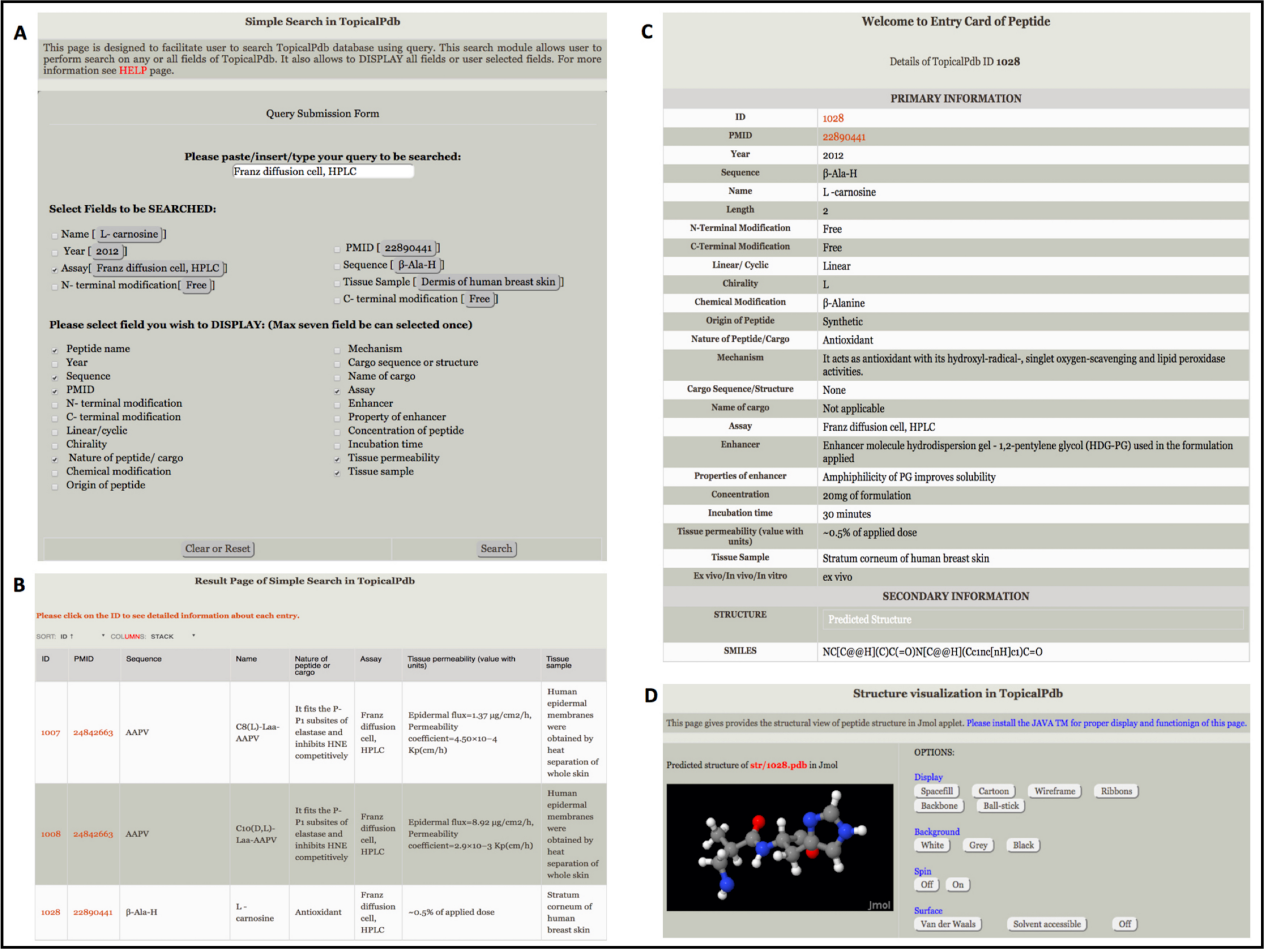
Screenshots of simple search module of TopicalPdb. **(A)** Simple search page allows users to search the database against a field of their choice, (B) result page of simple search, (C) Entry card with all fields of the peptide and (D) structure visualization of the peptide.

Simple search-This tool has been integrated to provide basic facility to search data from the database using a keyword (Fig 2). The users can perform a keyword search on any field of the database like PMID, name of the peptide, sequence of the peptide, assay, tissue sample and N- and C-terminal modifications. The users can select different fields to be displayed. The keyword should be without spaces.

Compounded search- This option facilitates complex search to extract desired information from TopicalPdb. It has multiple query system, by default it allows one to perform four queries at a time; user can perform search on any selected field. Server provides facility to use standard logical operators (e.g., =, >, <, and LIKE). User can combine output of different queries using operators like “AND & OR”. It also has option to add or remove queries to be executed.

#### Browsing tools

We have developed a browsing facility, which facilitates retrieval of information in a classified form. A user-friendly interface has been integrated for browsing peptides on major field’s that include conformation, peptide length and route of administration. User can browse topically delivered peptides using these fields. In addition, user can browse peptides based on their chemical modifications like N-terminal, C-terminal modifications and non-terminal modifications.

#### Analysis tools

TopicalPdb integrates various web-based tools for performing various analyses, like sequence similarity search, multiple sequence alignment, and structure alignment. We have integrated BLAST in TopicalPdb that allows users to perform BLAST search against peptides in the database. This allows users to identify topically delivered peptides in the database that have high sequence similarity with query peptide sequence. In addition to BLAST, we have also integrated another similarity search tools to perform sequence similarity based on Smith-Waterman algorithm. Peptide mapping tool allows users to identify peptides with the ability to be administered topically present within their proteins. The server searches such peptides and maps them on query protein submitted by the user. Multiple sequence alignment was implemented using HAlign[23]. HAlign employs parallel computing and center star strategy for fast and efficient alignment. Structural alignment was implemented using MUSCLE[24]. These alignment modules allow the users to align their query peptide sequence/structure with peptides present in TopicalPdb.

## Statistics and analysis of TopicalPdb

**Fig 3:**
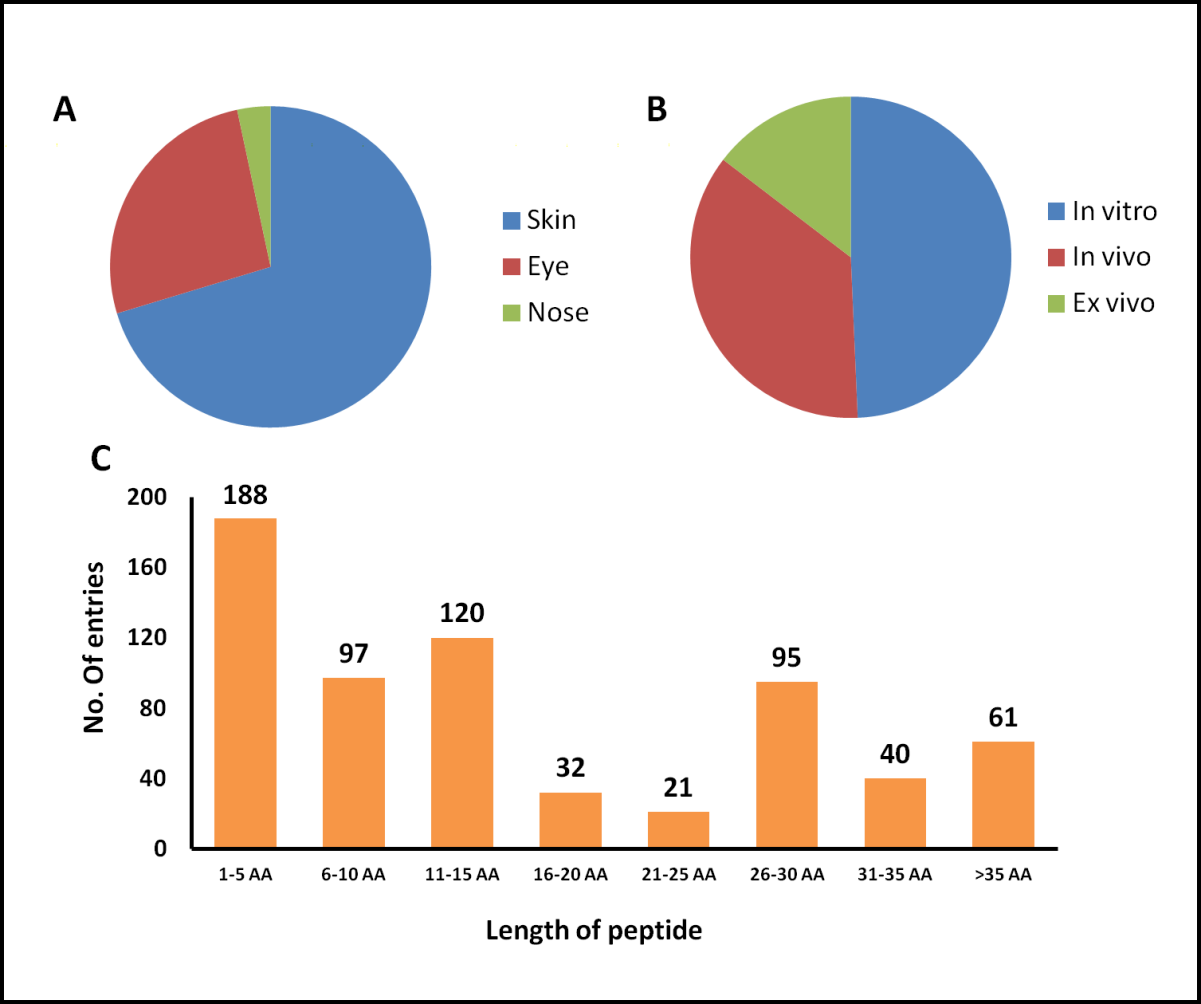
Distribution of peptides on the basis of (A) route of administration, (B) assay condition and (C) length of peptide.

TopicalPdb consists of 657 entries of experimentally validated topically delivered peptides. In the current version, we have compiled peptides, which have been shown to deliver through the skin, eye, and nose. Maximum entries were made of peptides, which were delivered by skin (462 entries) followed by eye (173 entries) and nose (22 entries) (Fig 3A). In TopicalPdb, most of the peptides are linear (584 entries) while a few peptides are cyclic (73 entries) in nature as well. Since the stability of the peptides always remains a matter of concern while developing peptide-based therapeutics. Therefore, various strategies like chemical modification, terminal modifications, insertion of D-amino acids, etc. have been used in order to enhance the stability of peptide therapeutics. We have compiled the information of any such modifications in peptide since it is very important to the users. In TopicalPdb, maximum entries (553) of the peptide consisting of L-amino acids while there are 85 entries where peptides contain both L-and D-amino acids. Only 2 entries were compiled where peptide are completely D-amino acids. In addition, a total 152 entries were compiled where peptides are modified at N-terminus like acetylation and 172 entries give information of peptides, which are modified at C-terminus like amidation. Besides, a total 189 entries were compiled with information of any other chemical modification like non-natural amino acid (e.g., β-Alanine).

Evaluation of skin-penetrating properties of peptides was carried out in different ways. Many peptides have been evaluated *in vitro* using Franz cell system as well as *in vivo* on mice skin. A few peptides have also been evaluated *ex vivo*. We have also compiled this information in TopicalPdb. A total 324 entries were compiled where peptide have been evaluated *in vitro*, while in 237 entries peptides were evaluated *in vivo*. A total 96 entries were made where peptides have been tested *ex vivo* (Fig 3B).

We have also analyzed the length of such peptides, which have been demonstrated to be delivered topically. It was found that peptides ranging from length 5–50 have been delivered topically but average length of such peptides is between 5 and 15 as around 400 entries contain peptides having length between 5 and 15 (Fig 3C).

**Fig 4:**
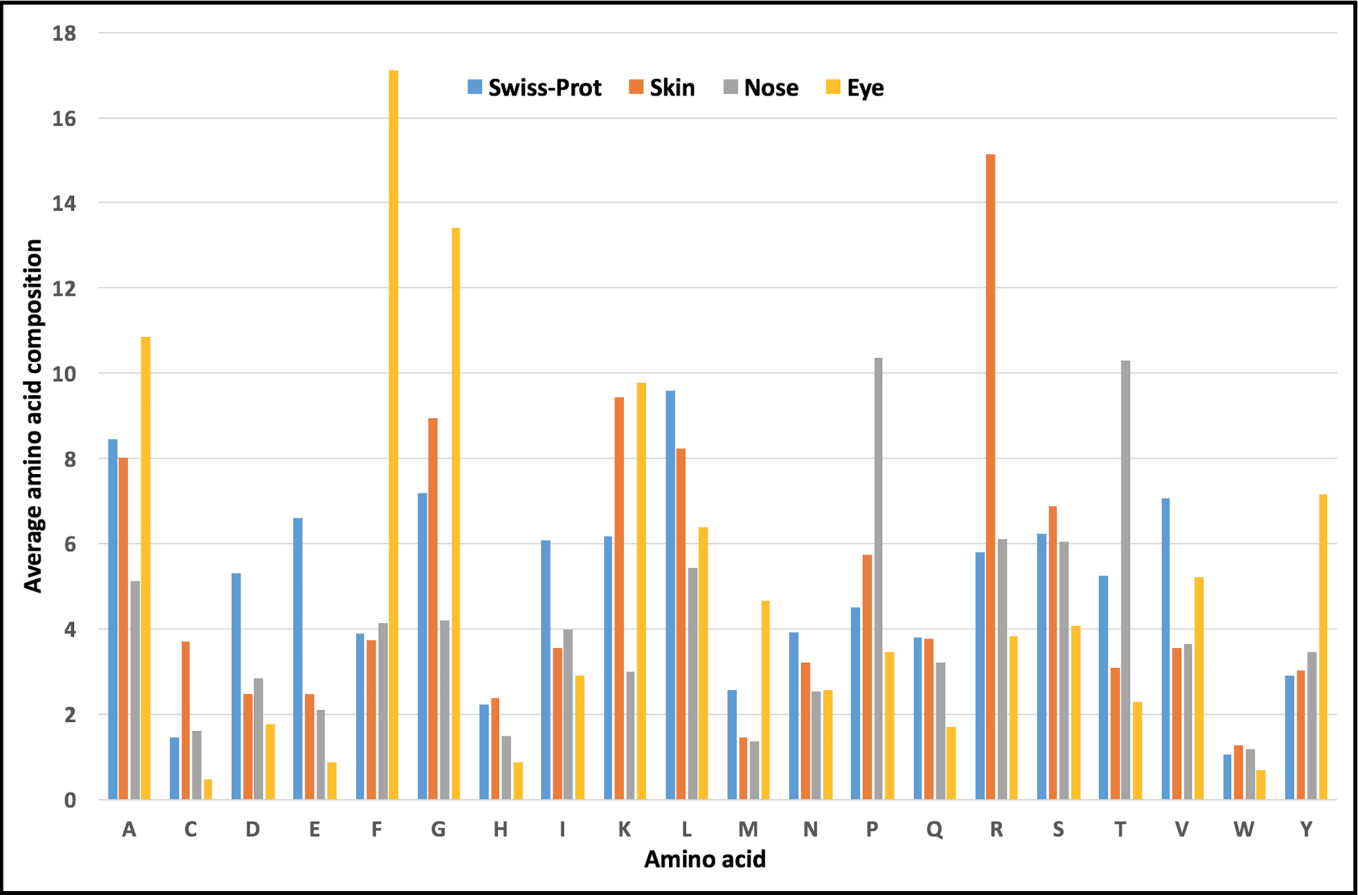
Amino acid composition of Swiss-Prot sequences and topically administered peptides through skin, nose and eye.

Fig 4 shows the comparison of the amino acid composition of different types of topical peptides in respect to Swiss-Prot sequences. It was observed that compared to Swiss-Prot, topically delivered peptides are depleted in negatively charged amino acids D and E and aliphatic amino acids like I, L and V. Peptides administered topically by the dermal route were found to enriched in R and S, while those administered through the nose were rich in P and T whereas A, F, G and Y were found to be enriched in peptides delivered topically through the eye.

To the best of authors’ knowledge, TopicalPdb is the first dedicated database of peptides, which have been delivered topically through skin eye and nose. With the integration of several analysis and browsing tools, with the integration of several analysis and browsing tools, TopicalPdb is a very useful resource to the researchers in several ways: (i) TopicalPdb provides comprehensive information of peptides which can penetrate the skin; thus, users can use these peptides to develop novel formulations with the existing drugs in order to enhance the efficacy of the drugs. Interfaces like search and browse help the researchers to fetch all possible peptides based on their queries. (ii) TopicalPdb also provides the structural information of peptides. This structural information is very useful for researchers and it can be exploited for docking studies with skin proteins to understand if there is any interaction of these proteins with peptides. It has been reported previously that a few skin-penetrating peptides interact with the skin proteins like keratin and this initial interaction leads to penetration of these peptides into the skin. Using structure of these peptides, user can screen peptides available in the TopicalPdb for potential interactions with skin proteins. (iii) SC is the outermost layer of skin and is the biggest hurdle for any substance to enter into the skin. Most of the drugs cannot penetrate this skin barrier and thus their efficacy is severely reduced. Therefore, understanding the properties of these peptides which are enabling them to cross this dead layer of skin, will certainly be helpful for the development of future topically administered drugs. As shown in Fig 4, there are certain amino acids which are found to be dominated in topically delivered peptides compared to other peptides. Using the information of these residues and motifs which actually responsible for skin-penetrating properties, users can design novel peptide sequences as well as can tweak the existing peptides for superior activity. Therefore, in this context, TopicalPdb also provides datasets for the analysis and development of computational prediction methods of skin penetrating peptides.

## Conclusion

In summary, TopicalPdb is a much-needed platform of topically delivered peptides providing comprehensive information of the peptides which are delivered through skin, eye and nose. In addition, TopicalPdb also gives information of various chemical modifications as well as structural information of these peptides, which makes it a useful resource for the scientific community working in the field of topical delivery of peptides.

## Limitations and update of TopicalPdb

In TopicalPdb, we have stored the predicted tertiary structures of most of the topically delivered peptides, even the peptides with modified or non-natural amino acids. However, the structure of few peptides having complex modifications have not been predicted due to the lack of the force-field libraries for such kind of residue modifications. In the future, we will try to predict the structure of such peptides.

One of the challenges in the field of database development is to update the information at regular intervals. Therefore, in this database, we have integrated an updating facility. Scientific community may submit TAPs to TopicalPdb using online submission form. Our team will update the information regularly from online submission as well as from literature.
